# Risk of traumatic brain injury among patients with ADHD and their unaffected siblings

**DOI:** 10.1038/s41390-024-03233-0

**Published:** 2024-05-07

**Authors:** Ping-Chung Wu, Shih-Jen Tsai, Ju-Wei Hsu, Kai-Lin Huang, Tzeng-Ji Chen, Tai-Long Pan, Mu-Hong Chen

**Affiliations:** 1 Department of Medical Education, Taipei Veterans General Hospital, Taipei, Taiwan; 2 Department of Psychiatry, College of Medicine, National Yang Ming Chiao Tung University, Taipei, Taiwan; 3 Department of Psychiatry, Taipei Veterans General Hospital, Taipei, Taiwan; 4 Department of Family Medicine, Taipei Veterans General Hospital, Taipei, Taiwan; 5 Institute of Hospital and Health Care Administration, National Yang Ming Chiao Tung University, Taipei, Taiwan; 6 Department of Family Medicine, Taipei Veterans General Hospital, Hsinchu Branch, Hsinchu, Taiwan; 7 School of Traditional Chinese Medicine, Chang Gung University, Taoyuan, Taiwan; 8 Research Center for Chinese Herbal Medicine and Research Center for Food and Cosmetic Safety, College of Human Ecology, Chang Gung University of Science and Technology, Taoyuan, Taiwan; 9 Liver Research Center, Chang Gung Memorial Hospital, Taoyuan, Taiwan

## Abstract

**Background:**

As the relationship between attention deficit hyperactivity disorder (ADHD) and traumatic brain injury (TBI) is gaining increasing attention, the TBI risk in patients with ADHD, unaffected siblings of ADHD probands, and non-ADHD controls remains unclear.

**Methods:**

Overall, 18,645 patients with ADHD, 18,880 unaffected siblings of ADHD probands, and 188,800 age-/sex-matched controls were followed up from enrollment to the end of 2011. The cases of TBI and TBI requiring hospitalization were identified during follow-up.

**Results:**

Patients with ADHD (hazard ratio [HR]: 1.57) and unaffected siblings (HR: 1.20) had an increased risk of any TBI compared with non-ADHD controls. Surprisingly, the likelihood of developing TBI requiring hospitalization during follow-up was higher in the unaffected siblings group (HR: 1.21) than in the control group, whereas it was lower in the ADHD probands group (HR: 0.86).

**Conclusions:**

Patients with ADHD and unaffected siblings of ADHD probands were more likely to develop any TBI during follow-up than controls. Unaffected siblings of patients with ADHD exhibited the highest risk of subsequent TBI requiring hospitalization compared with patients with ADHD and healthy controls. Therefore, TBI risk in patients with ADHD and their unaffected siblings would require further investigation.

**Impact:**

- ADHD diagnosis and ADHD trait are associated with risk of traumatic brain injury (TBI).
- Both patients with ADHD and their unaffected siblings were more likely to develop TBI during the follow-up compared with the control group.
- TBI requiring hospitalization occurred more in the sibling group than in the proband group.
- TBI risk should be closely monitored among unaffected siblings of patients with ADHD.

## Introduction

Attention deficit hyperactivity disorder (ADHD) is the most prevalent neurodevelopmental disorder among children, with an estimated prevalence increasing from 6.1% in 1997–1998 to 10.8% in 2022 according to the National Health Interview Survey of the U.S. population.^1,2^ ADHD often persists into adulthood and affects approximately 2.–3.4% of the adult population.^3,4^ Individuals with ADHD mostly have symptoms of inattention, hyperactivity, impulsivity, or a combination of these symptoms, which compromise basic functions and may adversely affect subsequent mental and physical health.^5–8^

Previous studies have demonstrated that both ADHD probands and their unaffected siblings tend to have impaired performance in a wide range of neuropsychological functions such as sustained attention and executive functions.^9–11^ Gau and Huang assessed attention performance in ADHD probands, unaffected siblings, and healthy controls using the Rapid Visual Information Processing (RVP) task of the Cambridge Neuropsychological Test Automated Battery (CANTAB). Subsequently, they discovered that probands with ADHD and their unaffected siblings had significantly higher total misses and a lower probability of hits in the RVP task than controls.^9^ Similarly, through analysis of the results of RVP-total hits, Pironti et al. found that cognitive impairments, especially in sustained attention, were present in both ADHD patients and their relatives.^10^ Increasing evidence has suggested that unaffected siblings of ADHD probands may exhibit a common endophenotype with their ADHD siblings and exhibit some deficits in attention, working memory, behavior inhibition, and executive functions.^12–14^

Traumatic brain injury (TBI), one of the major causes of death and disability in children, adolescents, and young adults globally, causes severe sequelae and burdens on the lives of patients, their families, and society.^15–17^ It has been estimated that more than 1.4 million people sustain a TBI each year in the United States, of whom 50,000 die from their injuries.^18^ Nguyen et al. performed a systematic review and meta-analysis of the global incidence of TBI and discovered a pooled incidence rate of 349 per 100,000 person-years for all ages.^19^ With its rapidly increasing prevalence and the increasing years of life lived with disability (YLDs) globally, TBI contributes considerably to the global injury burden.^20^

Several studies have suggested the potential relationship between ADHD and TBI.^21–23^ Ilie et al. conducted a cross-sectional study of 3,993 Canadian adults and observed significant positive associations between lifetime TBI and both current and past ADHD in this population.^21^ By comparing student athletes who had sustained a mild TBI with controls of similar age and sex, Biederman et al. reported that mild TBI subjects had a significantly higher rate of ADHD than controls, and that in all subjects, the onset age of ADHD was before the onset age of mild TBI.^22^ Furthermore, in a retrospective cohort study, Liou et al. demonstrated that patients with ADHD had a higher incidence of TBI than controls.^23^ However, few studies have investigated the likelihood of subsequent TBI development among ADHD patients, unaffected siblings of ADHD probands, and healthy controls. In a study consisting of 5,128 unaffected siblings of ADHD probands and 20,512 age- and sex-matched controls, Wei et al. observed that the unaffected siblings of patients with ADHD were more likely to develop TBI later in life compared with controls.^24^ The likelihood of subsequent TBI development among ADHD patients, unaffected siblings, and healthy controls is a topic worth exploring because the relationship between ADHD and TBI is gaining increasing attention, and the fact that unaffected siblings of ADHD probands may exhibit a common endophenotype and similar cognitive impairments with their ADHD siblings should be considered. Nevertheless, further studies and understanding of this topic seem to be limited. Furthermore, the effects of ADHD medications on the risk of subsequent TBI, the severity of subsequent TBI, and the age of TBI diagnosis have been rarely discussed in previous publications.

In this longitudinal, population-based cohort study, we investigated the risk of subsequent TBI, including skull fracture, concussion, contusion, and brain hemorrhage following injury, among patients with ADHD and their unaffected siblings and assessed the effect of ADHD medications on the risk of subsequent TBI, the severity of subsequent TBI, and the age of TBI diagnosis using the Taiwan National Health Insurance Research Database (NHIRD). We hypothesized that ADHD probands may have the highest risk of subsequent TBI, followed by unaffected siblings and controls during follow-up.

## Methods

### Data source

Taiwan NHIRD which consists of healthcare data from >99.7% of the entire Taiwan population is audited and released by National Health Research Institute for scientific and study purposes. In current study, we linked three databases together for the analysis. The first is the registry database for all beneficiaries (~28,000,000), which was used for the genealogy reconstruction and demographic characteristics based on Chen et al’s and Cheng et al’s methods.^25,26^ The second is the specialized dataset of mental disorders, which includes all medical (mental and non-mental) records between 2000 and 2011 of all insured individuals with mental disorders, and was used for the identification of ADHD probands. The third is the Longitudinal Health Insurance Database, which includes all medical records between 1996 and 2011 of 3,000,000 insured individuals that are randomly selected from entire Taiwanese people (~28,000,000), and was used for the identification of the unaffected siblings and control group. Individual medical records included in the NHIRD are anonymous to protect patient privacy. The diagnostic codes used were based on the International Classification of Diseases, 9th Revision, Clinical Modification (ICD-9-CM). The NHIRD has been used extensively in many epidemiologic studies in Taiwan.^25–28^ Institutional Review Board of Taipei Veterans General Hospital approved the study protocol and waived the requirement for informed consent since this investigation used de-identified data and no human subjects contact was required.

### Inclusion criteria for patients with ADHD, unaffected siblings and the control group

Patients who had a diagnosis of ADHD (ICD-9-CM code: 314) without prior TBI history between 2001 and 2010 were identified from the specialized dataset of mental disorders. To ensure diagnostic validity, a diagnosis of ADHD was given by board-certified psychiatrists at least twice (i.e., one psychiatrist at two different time points or two different psychiatrists) according to the clinical diagnostic interview and professional judgment. Individuals who had no ADHD diagnosis at any time in the database but had any sibling with ADHD were included as the ADHD sibling cohort (unaffected sibling group). The term “unaffected” sibling was probably a group that is not meeting the threshold for ADHD diagnosis compared with the identified ADHD probands but may share some of the underlying genetic as well as environmental risk factors for ADHD.^29–31^ The age-, sex-, birth time-, and residence-matched (1:10) control cohort was randomly identified from the Longitudinal Health Insurance Database after eliminating the study cases, those who had been given a diagnosis of ADHD at any time in the database, and those with any sibling with ADHD. Those who were diagnosed with TBI prior to January 01 2001 were excluded in the unaffected siblings and control group. The time of ADHD diagnosis was defined as the enrollment time in the ADHD proband group; January 01 2001 or the birthdate was defined as the enrollment time in the unaffected siblings and control groups. In addition, the long-term use of ADHD medications (methylphenidate or atomoxetine) during the follow-up was defined by the cumulative defined daily dose (cDDD) during the follow-up ≥ 365.^8^ The DDD recommended by the World Health Organization (WHO) Collaborating Center for Drug Statistics Methodology is a unit for measuring a prescribed amount of drug. The DDD is the assumed average maintenance dose per day of a drug consumed for its main indication. We calculated the sum of the dispensed DDD (cDDD) of ADHD medications during the follow-up period. Level of urbanization (level 1 to level 5; level 1: most urbanized region; level 5: least urbanized region) was also assessed for our study.^32^

### Main outcomes

TBI, including fracture of skull (ICD-9-CM codes: 800 ~ 801, 803 ~ 804), concussion (ICD-9-CM code: 850), contusion (ICD-9-CM code: 851), brain hemorrhage following injury (ICD-9-CM codes: 852, 853), and unspecified intracranial injury (ICD-9-CM codes: 854, 959.01), was identified during the follow-up (from enrollment to December 31 2011 or to the death). In addition, TBI requiring hospitalization was also identified.

### Statistical analysis

For between-group comparisons, the F test was used for continuous variables and Pearson’s X2 test for nominal variables, where appropriate. Cox regression models with adjustment of age, sex, residence and income were used to examine the hazard ratios (HRs) and 95% confidence intervals (CIs) of subsequent TBI and TBI requiring hospitalization in ADHD probands and unaffected siblings compared with the control group. Furthermore, we assessed the TBI and TBI requiring hospitalization likelihoods among ADHD probands with and without long-term use of ADHD medications, unaffected siblings, and control group. A 2-tailed *P* value of less than 0.05 was considered statistically significant. All data processing and statistical analyses were performed with Statistical Package for Social Science (SPSS) version 17 software (SPSS Inc.) and Statistical Analysis Software (SAS) version 9.1 (SAS Institute, Cary, NC).

## Results

In all, 18,645 patients with ADHD, 18,880 unaffected siblings of such patients, and 188,800 age-/sex-matched controls were included in our study. Patients with ADHD (4.48 ± 4.99 years) were younger than the other two groups (~ 6 years, *p* < 0.001) (Table 1). The ADHD proband group was male predominant (79.7%). During the follow-up, the ADHD probands had the highest incidence of developing any TBI (18.9%, *p* < 0.001), while the unaffected siblings had the highest incidence of developing TBI requiring hospitalization (1.5%, *p* = 0.005) (Table 1). TBI occurred younger in patients with ADHD (8.97 ± 6.15 years) and unaffected siblings (9.85 ± 7.24 years) than in the control (10.17 ± 7.59 years, *p* < 0.001) (Table 1). However, the TBI events took longer to occur in the ADHD proband group (4.56 ± 3.17 years) than in the other two groups (4.04 ± 2.84 years in unaffected siblings, 4.15 ± 2.88 years in the control group, *p* < 0.001) (Table 1).

**Table 1:** Demographic characteristics and TBI incidence among ADHD probands, unaffected siblings, and control group.

	A. ADHD probands (*n* = 18,645)	B. Unaffected siblings (*n* = 18,880)	C. Control group (*n* = 188,800)	*p* value	post-hoc
Age at enrollment (years, SD)	4.48 (4.99)	6.24 (6.39)	6.26 (6.41)	<0.001	A < B ~ C
Sex (n, %)				<0.001	
Male	14,857 (79.7)	8151 (43.2)	81,510 (43.2)		
Female	3788 (20.3)	10,729 (56.8)	107,290 (56.8)		
Level of urbanization (n, %)				<0.001	
1 (most urbanized)	3062 (16.4)	3534 (18.7)	35,340 (18.7)		
2	5705 (30.6)	5880 (31.2)	58,800 (31.2)		
3	1670 (9.0)	1780 (9.4)	17,800 (9.4)		
4	1623 (8.7)	1669 (8.8)	16,690 (8.8)		
5 (most rural)	6585 (35.3)	6017 (31.9)	60,170 (31.9)		
Income-related insured amount (n, %)				<0.001	
≤19,100 NTD/month	4974 (26.7)	3080 (16.3)	33,748 (17.9)		
19,001 ~ 42,000 NTD/month	6357 (34.1)	7086 (37.5)	75,533 (40.0)		
>42,000 NTD/month	7314 (39.2)	8714 (46.2)	79,519 (42.1)		
ADHD medications (n, %)					
< 365 cDDD	16,544 (88.7)				
≥365 cDDD	2101 (11.3)				
Incidence of TBI (n, %)	3525 (18.9)	2296 (12.2)	19,536 (10.3)	<0.001	A > B > C
Incidence of TBI requiring hospitalization (n, %)	209 (1.1)	281 (1.5)	2395 (1.3)	0.005	B > A ~ C
Age at TBI diagnosis (years, SD)	8.97 (6.15)	9.85 (7.24)	10.17 (7.59)	<0.001	A ~ B < C
Duration between enrollment and TBI (years, SD)	4.56 (3.17)	4.04 (2.84)	4.15 (2.88)	<0.001	A > B ~ C

*ADHD* attention deficit hyperactivity disorder, *TBI* traumatic brain injury, *SD* standard deviation, *NTD* New Taiwan dollars, *cDDD* cumulative defined daily dose.

**Fig. 1:**
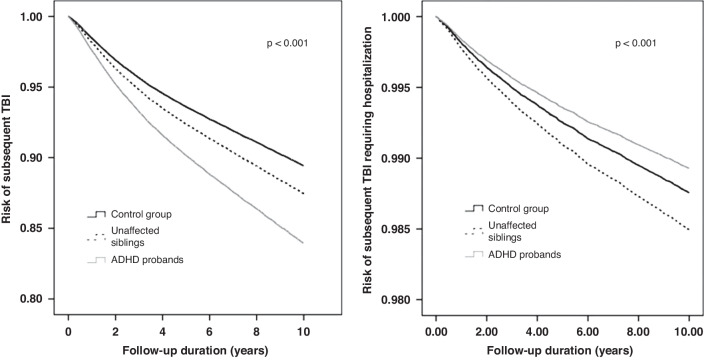
Risk of developing TBI and TBI requiring hospitalization between ADHD probands, unaffected siblings, and control group. ADHD attention deficit hyperactivity disorder, TBI traumatic brain injury.

Kaplan-Meier survival analyses with the log-rank tests of any TBI (*p* < 0.001) and TBI requiring hospitalization (*p* < 0.001) risks between groups were shown in the Fig. 1. The Cox regression models with full adjustment of age, sex, income, and level of urbanization showed that the patients with ADHD (HR: 1.57, 95% CI: 1.51–1.63) and the unaffected siblings of such patients (HR: 1.20, 95% CI: 1.15–1.25) were more likely to develop any TBI during the follow-up than the control group (Table 2). Surprisingly, the unaffected siblings (HR: 1.21, 95% CI: 1.07–1.37) were more likely, while the ADHD probands (HR: 0.86, 95% CI: 0.75–1.00) were less likely, to develop TBI requiring hospitalization during the follow-up compared with the control group (Table 2).

**Table 2:** TBI risk between ADHD probands, unaffected siblings, and control group.

	n (%)	HR	95% CI
TBI risk			
Control group	19,536 (10.3)	1 (ref.)	–
Unaffected siblings	2296 (12.2)	**1.20**	**1.15**–**1.25**
ADHD probands	3525 (18.9)	**1.57**	**1.51**–**1.63**
<365 cDDD	3145 (19.0)	**1.59**	**1.53**–**1.65**
≥365 cDDD	380 (18.1)	**1.44**	**1.30**–**1.60**
Risk of TBI requiring hospitalization			
Control group	2395 (1.3)	1 (ref.)	–
Unaffected siblings	281 (1.5)	**1.21**	**1.07**–**1.37**
ADHD probands	209 (1.1)	**0.86**	**0.75**–**1.00**
<365 cDDD	186 (1.1)	0.87	0.74–1.01
≥365 cDDD	23 (1.1)	0.84	0.55–1.25

Note: adjusting for age, sex, residence and income.

*ADHD* attention deficit hyperactivity disorder, *TBI* traumatic brain injury, *cDDD* cumulative defined daily dose.

Finally, the long-term use of ADHD medications may slightly reduce the risk of any TBI occurrence (HR: 1.44, 95% CI: 1.30–1.60) during the follow-up compared with the short-term use (1.59, 1.53–1.65) among patients with ADHD (Table 2).

## Discussion

The results of this large-scale, population-based study supported the hypothesis that ADHD probands had the highest risk of subsequent TBI during the follow-up period, followed by unaffected siblings and healthy controls. In addition, we observed a counter-intuitive result that unaffected siblings had a higher risk of TBI requiring hospitalization than did healthy controls, whereas ADHD probands had a lower risk than controls. Furthermore, our findings suggest that the use of ADHD medications may reduce the risk of subsequent TBI requiring hospitalization, and the results demonstrated an earlier mean age at TBI diagnosis in ADHD probands and unaffected siblings than in controls.

As mentioned in the introduction, the potential relationship between ADHD and TBI has been suggested in several studies.^21–23^ However, a meta-analysis comprising 3023 mild TBI patients and 9,716 controls revealed a significant association between ADHD and mild TBI, and the association was significant in studies that have reported on ADHD subsequent to mild TBI, but not in studies that have reported mild TBI subsequent to ADHD.^33^ As TBI can exacerbate attention and impulsivity problems,^34^ the relationship between ADHD and TBI could become bidirectional and complicated. Moreover, both ADHD and TBI are associated with a wide range of negative outcomes; thus, clarifying the temporal association is essential for developing effective prevention and treatment measures. In a prospective longitudinal study that examined the TBI diagnosis records of 628 male patients from birth to the age of 34 years, Guberman et al. discovered that childhood inattention-hyperactivity assessed using teacher rating scales was significantly associated with an increased risk of TBI from the ages of 11–34 years.^35^ Asarnow et al. conducted a meta-analysis of 12,374 patients with TBI of all severity levels and 43,491 controls and reported that 16.0% of patients with TBI presented with ADHD before brain injury; the prevalence of severe TBI was considerably higher than that of 10.8% reported for the general population.^36^ As increasing studies have supported the relationship between ADHD and subsequent TBI,^21–23,35,36^ our study discovered a similar mean duration of 4.56 years between study enrollment and subsequent TBI development among ADHD patients. Therefore, because ADHD appears to be a risk factor for TBI, more attention should be paid to this high-risk population. Although studying whether TBI is a risk factor for ADHD is a crucial topic, it is not addressed in this study.

According to previous studies that have assessed ADHD patients, unaffected siblings of ADHD probands, and healthy controls using psychiatric interviews and different executive function tasks, unaffected siblings of ADHD probands may exhibit a common endophenotype with their ADHD siblings, and they may also exhibit some deficits in a wide range of neuropsychological functions.^9–13^ In addition, several neuroimaging studies have examined neuroanatomical abnormalities in ADHD patients and their unaffected siblings.^10,37–40^ Pironti et al. found an abnormal decrease in the gray matter volume in the right inferior frontal gyrus and an abnormal increase in the white matter volume in the caudal portion of the right inferior fronto-occipital fasciculus among ADHD patients and their unaffected first-degree relatives.^10^ Hoogman et al. compared unaffected siblings with healthy controls and demonstrated shared familial effects by showing a significantly smaller surface area in the caudal middle frontal, lateral orbital frontal, and superior frontal gyrus in unaffected siblings.^37^ Chiang et al. reported increased functional connectivity in the left insula and left inferior frontal gyrus among both ADHD probands and unaffected siblings compared with controls.^38^ These studies have provided evidence that neural profiles are shared between ADHD patients and their unaffected siblings, and the results suggest that some of the shared neuroanatomical abnormalities may be associated with the severity of ADHD symptoms in unaffected siblings.^37,40^ To summarize, neuropsychological and neuroanatomical abnormalities found in ADHD probands and unaffected siblings may lead to the partial expression of ADHD symptoms and further adversely affect their subsequent mental and physical health.^6,7,24^ Although the relationship between ADHD and TBI has been examined in previous research,^21–23,33,35,36^ the association between TBI and unaffected siblings of ADHD probands has been rarely discussed. Wei et al. reported that unaffected siblings of ADHD probands were more likely to develop TBI (OR: 1.24, 95% CI: 1.14–1.36) than controls.^24^ Our study data also revealed this finding. Consequently, unaffected siblings and ADHD probands appear to be a high-risk population for TBI; thus, increasing the awareness of the increased TBI risk for their families and caregivers is warranted.

The severity of TBI has often been considered when exploring the relationship between TBI and subsequent ADHD.^33,36,41^ However, fewer studies have considered the severity of TBI because more studies are focusing on the association between ADHD and subsequent TBI development. In this study, we also considered the severity of TBI by identifying TBI requiring hospitalization. The results revealed that when compared with controls, unaffected siblings were at a higher risk of TBI requiring hospitalization, whereas ADHD probands were at a lower risk. By using the German Pharmacoepidemiological Research Database, Lindemann et al. performed a large-scale retrospective cohort study to assess the risk of hospitalization due to injury diagnoses in children and adolescents with newly diagnosed ADHD compared with those without ADHD,^42^ and the results were different from our findings. Moreover, they reported that the incidence of TBI hospitalization was 1.87% (95% CI: 1.71–2.04) in male ADHD patients, 1.32% (95% CI: 1.19–1.47) in male controls, 1.38% (95% CI: 1.15–1.65) in female ADHD patients, and 0.91% (95% CI: 0.73–1.13) in female controls, with an increased adjusted HR of TBI hospitalization for patients with ADHD compared with those without. By contrast, our study reported that the incidence of TBI requiring hospitalization was 1.1% in ADHD patients and 1.3% in healthy controls, with a decreased adjusted HR of TBI requiring hospitalization for ADHD probands compared with controls. To explain the differences, we proposed the following possible reasons. The differences may be attributed to some biological, psychological, and sociological factors that were not investigated but could influence the incidence rates. For instance, Lindemann et al. did not consider the ADHD medication treatment as a possible protective factor against TBI in ADHD patients; however, the factor was discussed further in the study. Consequently, the results are potentially confounded by the fraction of patients receiving ADHD medications. In addition, other unmeasured potential prognostic factors, such as socioeconomic status, psychological stress, and environmental safety, may influence the results and contribute to the differences. Due to the limited relevant literature, further studies are required to reveal more details. Moreover, our study discovered a counter-intuitive and surprising result that ADHD probands had a decreased risk of TBI requiring hospitalization compared with healthy controls, which might be because ADHD probands may be in a safer setting, be under more protection, and receive more care from caregivers given their diagnosis of ADHD than other healthy individuals. The aforementioned reasons may also explain our finding of the longest duration between enrollment and TBI occurrence in the ADHD probands compared with the other two groups. Nevertheless, these measures to prevent ADHD patients from physical injuries have some limitations because of the increased TBI risk among ADHD patients shown in this study, but they may play an important role in reducing their risk of TBI hospitalization. The beneficial effect of the use of ADHD medications on subsequent TBI requiring hospitalization could also explain the difference. However, the result should be interpreted with caution, and further studies are needed to confirm the hypothesis. Additionally, our study reported that the unaffected siblings of ADHD probands were more likely to develop both TBI and TBI requiring hospitalization than healthy controls. Hence, we recommend that more attention be paid to preventing subsequent TBI development in this population.

Previous studies have revealed that ADHD medications are related to decreases in the risks of a wide range of ADHD-associated functional outcomes, including TBI and accidents and injuries.^23,43–47^ Mikolajczyk et al. demonstrated a 34% risk reduction for hospitalization due to brain injury diagnoses during the periods with ADHD medication compared with nonmedicated periods.^45^ Liou et al. examined 72,181 ADHD patients and 72,181 age-and sex-matched non-ADHD controls and reported the association between the long-term use of ADHD medications and a reduced risk of subsequent TBI.^23^ Boland et al. conducted a meta-analysis of 21 studies in 2020 and revealed a robust protective effect of ADHD medications on academic outcomes, accidents and injuries, and mood disorders, along with a statistically insignificant protective effect on TBI.^46^ More recently, Brunkhorst-Kanaan et al. conducted a systematic review and suggested that stimulant medication appeared to be effective for injury prevention in ADHD patients over their lifespan.^47^ Compatible with the results of previous studies, our study results suggest that the use of ADHD medications may reduce the risk of subsequent TBI requiring hospitalization. Therefore, early diagnosis and optimal treatment for individuals with ADHD are critical in clinical practice to minimize the risk of subsequent TBI requiring hospitalization. In addition, further well-designed clinical studies may be needed to quantify the protective effects of ADHD medications on subsequent TBI risk.

Age at TBI diagnosis may influence neurocognitive, academic, and behavior outcomes following TBI and is a topic worth exploring.^48–53^ However, previous studies have reported mixed results. Some studies have supported the theory of neuroplasticity describing that children injured at an earlier age have better outcomes due to the ability for neuronal circuits in the young brain to undergo adaptive changes on structural and functional levels,^51,52^ whereas other studies have supported the theory of vulnerability stating that children injured at an earlier age have poorer outcomes because of the incomplete development of the brain after TBI.^48–50,53^ The impact of age at TBI diagnosis on the results is difficult to assess because of the increasing difficulty of detecting neurocognitive impairments in younger children, the lack of large-scale studies, and variations in the distribution of age categories and the timing of follow-up evaluations in various studies.^54,55^ In this study, we collected a wide range of demographic data on age at TBI diagnosis from a considerably large-sized sample. In addition, we observed an earlier mean age at TBI diagnosis in ADHD probands and unaffected siblings compared with controls. However, the impact of age at injury on outcomes following TBI remains controversial; therefore, further studies are necessary to corroborate this finding.

However, this study has several limitations. First, the incidence of TBI and TBI requiring hospitalization may have been underestimated because only those who seek medical help are registered in the NHIRD. Second, information on the severity of ADHD and TBI is unavailable in the NHIRD. Therefore, we did not account for the severity of ADHD and identified TBI requiring hospitalization as an alternative way to assess the severity of TBI. Further studies would be required to clarify the association of ADHD diagnosis and trait with the exact severity, which was defined by the TBI neuroimaging criteria and Glasgow Coma Scale, of TBI. Finally, because the NHIRD does not provide information on factors such as psychosocial stress, family relationships, personal lifestyle, and environment, we could not explore their influence. Therefore, these limitations should be considered when interpreting the results.

In conclusion, this large-scale, population-based study suggested that ADHD probands had the highest risk of subsequent TBI, followed by their unaffected siblings and controls. We also found that unaffected siblings had a higher risk of TBI requiring hospitalization than controls, whereas ADHD probands had a lower risk. Hence, we recommend that more attention should be paid in order to prevent subsequent TBI development in the unaffected siblings of ADHD probands. In addition, this study demonstrated that the use of ADHD medications may reduce the risk of subsequent TBI requiring hospitalization, which supports the importance of early diagnosis and optimal treatment for individuals with ADHD.

## Data Availability

The NHIRD was released and audited by the Department of Health and Bureau of the NHI Program for the purpose of scientific research (https://www.apre.mohw.gov.tw/). The NHIRD can be accessed through a formal application that is regulated by the Health and Welfare Data Science Center of Ministry of Health and Welfare, Taiwan.
